# Implementation of a Multi-Disciplinary Team and Quality of Goals of Care Discussions in Palliative Surgical Oncology Patients

**DOI:** 10.1245/s10434-023-14190-z

**Published:** 2023-09-06

**Authors:** Joel J. Y. Soon, Darryl W. K. Juan, Whee S. Ong, Schin Bek, Patricia S. H. Neo, Ennaliza Salazar, Kun Da Zhuang, Yee Pin Tan, Chin Jin Seo, Johnny C. A. Ong, Claramae S. Chia, Jolene S. M. Wong

**Affiliations:** 1 Division of Surgery and Surgical Oncology, Singapore General Hospital, Singapore, Singapore; 2 Department of Sarcoma, Peritoneal and Rare Tumours (SPRinT), Division of Surgery and Surgical Oncology, National Cancer Centre Singapore, Singapore, Singapore; 3 Department of Sarcoma, Peritoneal and Rare Tumours (SPRinT), Division of Surgery and Surgical Oncology, Singapore General Hospital, Singapore, Singapore; 4 Division of Clinical Trials and Epidemiological Sciences, National Cancer Centre Singapore, Singapore, Singapore; 5 Yong Loo Lin School of Medicine, National University of Singapore, Singapore, Singapore; 6 Division of Supportive and Palliative Care, National Cancer Centre Singapore, Singapore, Singapore; 7 Department of Gastroenterology and Hepatology, Singapore General Hospital, Singapore, Singapore; 8 Vascular and Interventional Radiology, Singapore General Hospital, Singapore, Singapore; 9 Department of Psychosocial Oncology, National Cancer Centre Singapore, Singapore, Singapore; 10 Duke-NUS Medical School, SingHealth Duke-NUS Oncology Academic Clinical Program, Singapore, Singapore; 11 Duke-NUS Medical School, SingHealth Duke-NUS Surgery Academic Clinical Program, Singapore, Singapore; 12 Laboratory of Applied Human Genetics, Division of Medical Sciences, National Cancer Centre Singapore, Singapore, Singapore; 13 Institute of Molecular and Cell Biology, A*STAR Research Entities, Singapore, Singapore

## Abstract

**Background:**

Palliative surgical oncology patients represent a unique group with complex needs who often require multidisciplinary input for the provision of timely and holistic care. The authors assembled a multi-disciplinary palliative intervention team and evaluated its association with the quality of discussions on goals of care (GOC) among advanced cancer patients undergoing palliative interventions.

**Methods:**

This prospective cohort study analyzed advanced cancer patients undergoing palliative interventions at a single urban academic center from October 2019 to March 2022. In January 2021, a multi-disciplinary palliative surgical intervention (MD-PALS) team was assembled. All palliative surgical oncology patients were discussed at multi-disciplinary meetings and managed by members of the MD-PALS team. An interrupted time series (ITS) model was built to evaluate the association of MD-PALS implementation and the quality of GOC discussions as measured by a consensus-derived four-point GOC discussion quality score.

**Results:**

The study recruited 126 palliative surgical oncology patients: 44 in the pre-MD-PALS group and 82 in the post-MD-PALS group. The two groups did not differ significantly in baseline demographics, treatment, or postoperative and survival outcomes. Compared with the pre-MD-PALS group, the post-MD-PALS group had a significantly higher mean GOC discussion quality score (1.34 vs 2.61; *p* < 0.001). Based on the ITS model, the average quarterly GOC discussion quality score increased significantly among patients after implementation of the MD-PALS team (change = 1.93; 95 % confidence interval, 0.96–2.90; *P* = 0.003).

**Conclusion:**

The implementation of an MD-PALS team was associated with improvements in the quality of GOC discussions among palliative surgical oncology patients.

**Supplementary Information:**

The online version contains supplementary material available at 10.1245/s10434-023-14190-z.

Advanced cancer is prevalent in an ageing population and accounts for up to 30 % of deaths in many countries.^1^ In the recent decade, palliative interventions have been increasingly used during end-of-life (EOL) situations for surgical oncology patients to reduce symptoms and improve quality of life (QoL).^2,3^ However, less than 10 % of surgical patients receive specialist palliative care consultations or have goals-of-care (GOC) discussions within the last year of life.^4^ Furthermore, advanced cancer patients often are faced with complex oncologic issues due to the interplay of systemic, locoregional treatment options and disease biology, complicating decision-making processes near EOL.

Although an inter-disciplinary approach with conventional tumor boards involving surgical, medical, and radiation oncologists represents the standard of care in the management of routine surgical oncology patients, it may be suboptimal in the setting of palliative surgical oncology.^5,6^ Palliative patients often are plagued with unique EOL issues such as symptom control, emotional distress, social support, and spirituality issues.^7,8^

To address this uniquely vulnerable population, we established a multi-disciplinary palliative surgical intervention (MD-PALS) team comprising specialist palliative care physicians, surgical oncologists, medical and radiation oncologists, gastroenterologists, nutritionists, interventional radiologists, rehabilitation physicians, psychiatrists, psychologists, and medical social workers. We hypothesized that the diversity and experience of MD-PALS members can facilitate and improve care delivery for palliative surgical oncology patients with complex EOL needs. In this study, we aimed to evaluate the relationship between the implementation of MD-PALS and the quality of GOC discussions among advanced cancer patients and their families during surgical admissions.

## Methods

### Study Design and Population

We performed a single-center prospective cohort study of advanced cancer patients who received palliative interventions (i.e., surgery, endoscopic or interventional radiologic procedures) at the Division of Surgery and Surgical Oncology of the Singapore General Hospital between October 2019 and March 2022. This study was approved by the SingHealth Centralized Institutional Review Board, and informed consent obtained before enrolment of patients.

### Intervention: MD-PALS Team

The MD-PALS team, assembled in January 2021, included members from surgical oncology, medical and radiation oncology, palliative care physicians, gastroenterologists, interventional radiologists, advanced practice and specialty nurses, nutritionists, psychologists, and medical social workers. Fortnightly meetings were held by the MD-PALS team to discuss all patients receiving palliative interventions from January 2021 onward. Before assembly of the MD-PALS team, specialist consultations from the respective members of the group were requested on an ad hoc basis by the primary team caring for the patient.

Advanced cancer patients who received palliative interventions between October 2019 and December 2020 were in the pre-MD-PALS group, whereas those admitted between January 2021 and March 2022 were in the post-MD-PALS group.

### Outcome Measure: Quality of Discussions on GOC

The primary outcome of the study was the quality of GOC discussions, which was measured using a four-point composite score as follows: 1 (intent and expected benefits and morbidities associated with palliative surgery or other interventions), 2 (conveyance of overall prognosis), 3 (consideration of patients’ priorities and goals of treatment), 4 (determination of code status). A score of 0 or 1 was assigned if the aforementioned components were respectively absent or present from clinical documentation, with a maximum possible GOC discussion quality score of 4 points. These components were reviewed and considered essential in GOC discussions during a consensus meeting held among the MD-PALS team members. Communication documentation review was performed by two physicians (J.J.Y.S. and J.S.M.W.) independently, and conflicts were resolved by a third senior author (C.S.M.C.).

We also reviewed the number of consultations by specialist palliative care physicians and other members of the MD-PALS team during each patient’s index surgical admission for palliative intervention.

### Other Study Covariates

Data on patient demographics, tumor characteristics, palliative interventions, postoperative or postprocedural complications, length of index admission, systemic chemotherapy or radiotherapy, and hospital readmission were collected via manual chart review of electronic medical records at the index surgical admission for palliative intervention.^9^

### Statistical Analysis

Continuous characteristics were compared using the two-sample *t* test, whereas categorical characteristics were compared using Fisher’s exact test. Overall survival (OS) was measured from the date of palliative intervention to the date of death. Alive patients were censored as of 11 August 2022, when data were cut off for analysis, and no patients were lost to follow-up evaluation.

The Kaplan-Meier method was used to estimate OS distribution, and the log-rank test was used to compare OS between the two patient groups. Because the longer follow-up duration among the pre-MD-MPALS patients could distort the OS comparison, a sensitivity analysis was performed in which the OS duration of all patients who survived beyond 12 months was censored at 12 months.

Mean GOC discussion quality scores between the pre- and post-MD-PALS groups were compared using the two-sample *t* test, with additional adjustments made for age, Eastern Cooperative Oncology Group (ECOG) performance status, and whether the patient underwent palliative surgery using analysis of covariance. A segmented linear regression model was fitted to evaluate the change in the level and trend of the average quarterly GOC discussion quality score after MD-PALS implementation. Diagnostic checks of the fitted model were performed to ensure that all model assumptions were met.

To evaluate the robustness of the results from this interrupted time series (ITS) analysis, a sensitivity analysis based on segmented beta regression of the mean proportion of quality components of GOC discussions achieved by the patients was performed.

Exploratory subgroup analyses were performed to examine the differences in GOC discussion quality scores between the pre- and post-MD-PALS groups by type of palliative interventions using logistic regression. Firth’s penalized likelihood estimation method was used for covariates with a complete separation issue. Goodness of fit was assessed based on the Hosmer-Lemeshow test.

All statistical tests were two-sided with a 5 % significance level. Analyses were performed using SAS 9.4 (SAS Institute Inc., Cary, NC, USA) and Stata 16.1 (StataCorp, College Station, TX, USA).

## Results

The study enrolled 44 (34.9 %) patients in the pre-MD-PALS group and 82 (65.1 %) patients in the post-MD-PALS group. The median follow-up period was 27.1 months in the pre-MD-PALS group and 10.6 months in the post-MD-PALS groups. The two groups did not differ significantly in terms of clinical and demographic characteristics (Table 1). The most common indication for consideration of palliative interventions was intestinal obstruction. After intervention, the pre- and post-MD-PALS groups did not differ significantly in hospital readmissions (50.0 % vs 47.6 %; *P* = 0.853), and the median overall survival (OS) durations were respectively 9.2 and 5.8 months (*P* = 0.410).

**Table 1:** Baseline and clinical characteristics of advanced cancer patients undergoing palliative interventions before (October 2019 to December 2020) and after (January 2021 to March 2022) implementation of a multi-disciplinary palliative intervention (MD-PALS) team

	Pre-MD-PALS	Post-MD-PALS	*P* value
	(*n* = 44)* n* (%)	(*n* = 82) *n* (%)	
Mean age at consideration for palliative intervention (years)			
Mean (SD)	64 ± 12.9	63 ± 11.7	0.86
Gender			0.17
Male	19 (43.2)	24 (29.3)	
Female	25 (56.8)	58 (70.7)	
Ethnicity			0.20
Chinese	36 (81.8)	60 (73.2)	
Malay	2 (4.5)	10 (12.2)	
Indian	5 (11.4)	5 (6.1)	
Others	1 (2.3)	7 (8.5)	
Marital status			0.27
Single	6 (13.6)	20 (24.4)	
Married	27 (61.4)	49 (59.8)	
Divorced/separated	11 (25.0)	12 (14.6)	
Unknown	0	1 (1.2)	
ECOG performance status			0.59
0	10 (22.7)	16 (19.5)	
1	23 (52.3)	36 (43.9)	
2	8 (18.2)	24 (29.3)	
3	3 (6.8)	6 (7.3)	
Primary cancer			0.80
Colorectal/appendiceal	20 (45.5)	38 (46.3)	
Gastric	5 (11.4)	6 (7.3)	
Ovarian/gynecologic	12 (27.3)	27 (32.9)	
Others	7 (15.9)	11 (13.4)	
Lines of chemotherapy before study entry			0.18
None	21 (47.7)	27 (32.9)	
1	11 (25.0)	16 (19.5)	
2	6 (13.6)	18 (22.0)	
≥ 3	6 (13.6)	21 (25.6)	
Reason for consideration of palliative intervention^a^			0.055
Intestinal obstruction	37 (84.1)	55 (67.1)	
Bleeding	2 (4.5)	2 (2.4)	
Pain	2 (4.5)	1 (1.2)	
Sepsis	2 (4.5)	10 (12.2)	
Fistula	0	6 (7.3)	
Others	1 (2.3)	8 (9.8)	
Type of palliative intervention			0.063
Surgery	42 (95.5)	67 (81.7)	
Endoscopy	2 (4.5)	8 (9.8)	
IR procedures	0	7 (8.5)	
Postoperative complications^b^			0.082
None	39 (88.6)	58 (70.7)	
Minor (grade I or II)	3 (6.8)	15 (18.3)	
Major (Grade III or IV)	2 (4.5)	9 (11.0)	
Mean length of index admission (days)			
Mean (SD)	20.6 ± 14.2	24.1 ± 19.0	0.24
Ongoing chemotherapy	16 (36.4)	26 (31.7)	0.69
Ongoing radiotherapy	3 (6.8)	1 (1.2)	0.12
Median overall survival: months (95 % CI)			
	9.2 (4.6–16.4)	5.8 (3.5–8.8)	0.41
Any hospital readmissions	22 (50.0)	39 (47.6)	0.85

*MD-PALS* multi-disciplinary palliative surgical intervention, *ECOG* Eastern Cooperative Oncology Group, *IR* interventional radiologic, *CI* confidence interval

^a^16 Patients had additional reason for consideration of palliative intervention

^b^Based on Clavien-Dindo classification^10^

The mean GOC discussion quality score was significantly higher in the post-MD-PALS group than in the pre-MD-PALS group (1.34 vs 2.64; *P* < 0.001; Table 2). After adjustment for age, ECOG performance status, and whether the patients received palliative surgery, the mean GOC discussion scores remained significantly higher in the pre-MD-PALS group than in the post-MD-PALS group (1.34 vs 2.61; *P* ≤ 0.001). The proportion of patients who received quality discussions on goals of surgery, prognosis, priorities and preferences of treatment options, and code status increased significantly after MD-PALS implementation (all *P* < 0.05; Table 3). Subgroup analysis of GOC discussion quality scores by palliative intervention types did not show differences for the patients who underwent surgical, endoscopic, or radiologic interventions (Table S2).

**Table 2:** Composite score of quality of palliative care

	Pre-MD-PALS		Post-MD-PALS		*P* value
	*n*	%	*n*	%	
Total	44	100.0	82	100.0	
Score					
0	10	22.7	0	–	< 0.001
1	13	29.5	12	14.6	
2	17	38.6	25	30.5	
3	4	9.1	28	34.1	
4	0	–	17	20.7	
Mean score	1.34 ± 0.94		2.61 ± 0.98		< 0.001
After adjustment for covariates^a^					
Mean score (SE)	1.48 (0.19)		2.70 (0.14)		< 0.001

*MD-PALS* multi-disciplinary palliative surgical intervention, *SE* standard error

^a^Age, Eastern Cooperative Oncology Group (ECOG) performance status, and palliative intervention type

**Table 3:** Components of preoperative discussion on goals of care

	Pre-MD-PALS	Post-MD-PALS	*P* value
	(*n* = 44) *n* (%)	(*n* = 82)* n* (%)	
Deliberations on goals of surgery	33 (75.0)	82 (100.0)	< 0.001
Deliberations on patient’s prognosis	13 (29.5)	45 (54.2)	0.009
Deliberations on patient’s priorities and preferences on treatment options	12 (27.3)	69 (84.1)	< 0.001
Resuscitation code status discussions	1 (2.3)	18 (22.0)	0.003

*MD-PALS* multi-disciplinary palliative surgical intervention

**Fig. 1:**
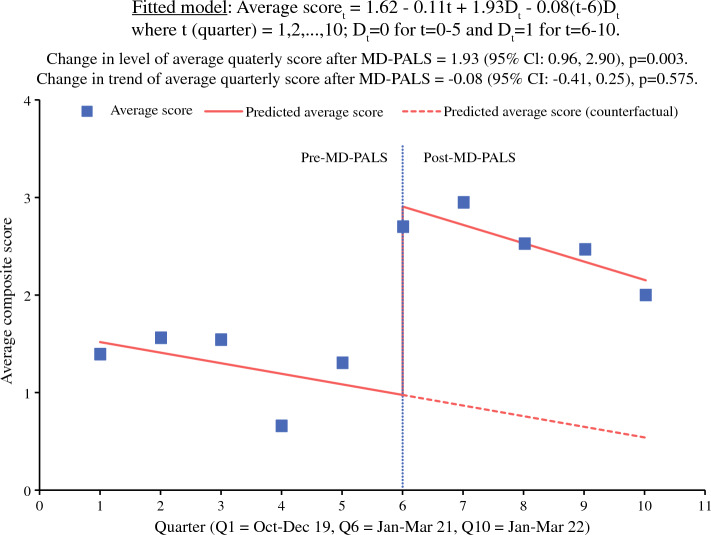
Interrupted time series analysis of the average quarterly composite score of quality of goals of care discussions.

The interrupted time series (ITS) analysis showed a significant increase in the average quarterly GOC discussions quality score by 1.93 points (95 % conficence interval [CI], 0.96–2.90; *P* = 0.003) after MD-PALS implementation (Fig. 1). The trend of the average quarterly GOC discussions quality score however remained the same as that during the pre-MD-PALS era. The conclusions on the changes in the level and trend of the average GOC discussions quality score after MD-PALS implementation based on the sensitivity ITS analysis were similar to those of the original ITS analysis. Details of this sensitivity ITS analysis are presented in Fig. S1 and Table S1.

A higher proportion of the post-MD-PALS patients than the pre-MD-PALS patients had specialist palliative care consultations (41.5 % vs 31.8 %; *P* = 0.339) and inputs from the various members of the multi-disciplinary teams (84.1 % vs 81.8 %; *P* = 0.804) during their index admission for palliative interventions, although these differences did not reach statistical significance (Table 4).

**Table 4:** Specialist palliative care consultations and multi-disciplinary team inputs during index hospital admission

	Pre-MD-PALS	Post-MD-PALS	*P* value
	(*n* = 44) *n* (%)	(*n* = 82) *n* (%)	
Specialist palliative care consultations	14 (31.8)	34 (41.5)	0.34
Input from multi-disciplinary team	36 (81.8)	69 (84.1)	0.80
Input from medical oncology^a^	20/38 (52.6)	46/80 (57.5)	0.69
Input from radiation oncology^a^	3/20 (15.0)	11/70 (15.7)	1.00
Input from TPN/nutrition team^a^	15/30 (50.0)	43/75 (57.3)	0.52
Input from psychology oncology/medical social worker^a^	11/31 (35.5)	35/79 (44.3)	0.52
Input from vascular & interventional radiology^a^	23/32 (71.9)	44/78 (56.4)	0.20
Input from gastroenterology^a^	29/42 (69.0)	21/71 (29.6)	< 0.001

*MD-PALS* multi-disciplinary palliative surgical intervention, *TPN* total parenteral nutrition

^a^Only among applicable patients

## Discussion

Overall, findings from our study suggest that the implementation of a multi-disciplinary palliative intervention team (MD-PALS) and the involvement of the various specialist providers in the care of palliative surgical oncology patients improved one aspect of palliative care delivery: the quality of GOC discussions conducted. This may be attributable to increased collaboration and a ready exchange of clinical information as a result of the MD-PALS platform. The primary surgical oncologist, informed by MD-PALS members, can now better identify key EOL issues surrounding the palliative surgical patient and is well-placed to conduct quality GOC discussion during a surgical admission.

In previous efforts to improve the quality of GOC discussions, investigators have evaluated the use of didactic lectures, one-on-one training, or even role-playing with standardized patients.^10–15^ Although these are important in equipping clinicians with the skills necessary to conduct GOC discussions, their effectiveness may be limited by varying levels of proficiency among the members of a multidisciplinary team and differences in opinions regarding the most goal-concordant management plan. In our anecdotal experience, it is not uncommon for patients to have encounters with various members of the multidisciplinary team, with a resulting lack of clarity in terms of their GOC and management plan. Therefore, the MD-PALS team was formed at our institution to build a “Community of Practice” aimed at improving palliative care processes, enhancing interdisciplinary collaboration, and streamlining communication with patients and their families.^16^

Increased involvement of specialist palliative care physicians in the care of palliative surgical oncology patients during the post-MDPALS era (31.8 % vs 41.5 %) also likely contributed to the improvements in the quality of GOC discussions. A randomized controlled trial by Vanbutsele et al.^17^ demonstrated that early and systematic integration of palliative care improves the quality of life and is more beneficial for patients with advanced cancer than palliative care consultations offered on demand. The authors concluded that the results may be attributable to the different focus of treatment offered by the palliative team compared with the oncology team. In our experience, the specialist palliative care team at our institution contributed significantly in terms of integrating the recommendations of MD-PALS and facilitating the GOC discussions with the patients and families.

The current study was limited by the relatively small sample and the unequal cohort sizes of the pre- and post-MD-PALS groups. We hypothesized that the smaller pre-MD-PALS cohort was likely due to changes in workflows and elective hospital admissions during the COVID-19 pandemic.^18–21^ Because the pre-MDPALS era coincided with the outbreak of COVID-19 in Singapore, the lower proportion of patients and families receiving GOC discussions also may be explained by isolation and visiting policies. In addition, there is no existing international consensus on what constitutes quality GOC discussions, and our MD-PALS consensus definition of the four essential GOC components informed by literature and our local clinical experience may limit the generalizability of this study.^22–24^

In conclusion, the implementation of an MD-PALS team improved the quality of GOC discussions among palliative surgical oncology patients. These findings provide an opportunity and direction for future studies on improving the quality of palliative care of advanced cancer patients.

### Supplementary Information

Below is the link to the electronic supplementary material.

Supplementary file1 (DOCX 14 kb)

Supplementary file2 (DOCX 13 kb)

Supplementary file3 (DOCX 17 kb)

Supplementary file4 (JPG 17 kb)
