# Supplementary material for: Implementation of a Multi-Disciplinary Team and Quality of Goals of Care Discussions in Palliative Surgical Oncology Patients
Source: Ann Surg Oncol. 2023 Sep 6;30(13):8054–60. doi: 10.1245/s10434-023-14190-z (PMC10625938; doi:10.1245/s10434-023-14190-z)
Supplement: Supplementary file 1 — Supplementary file1 (DOCX 14 kb) [file 10434_2023_14190_MOESM1_ESM.docx]

Supplementary Material: Robustness check on the interrupted time series (ITS) analysis of average quarterly composite score of goals of care (GOC) conversations

The segmented linear regression of average quarterly composite score of GOC conversations was based on a simple model and its ITS plot clearly communicated the effects of the implementation of the MD-PALS team on the quality of GOC conversations. Though this fitted model fulfilled all diagnostic checks, there was a concern of model overfitting given that the model was built on a small sample size of just 10 data points. We fitted additional alternative ITS models to evaluate the robustness of the results from this linear regression model.

We first repeated the linear regression model fitting process on the time series of average monthly composite score. However, this model failed diagnostic checks.

Next, we transformed the composite score into a proportion by dividing the score of each patient by 4. In order to fit a beta regression model, we applied a further transformation suggested by Smithson and Verkuilen on the proportions so that the final transformed values excluded the extreme values of 0 and 1 as required by the beta distribution^1^. The fitted beta regression model for the mean proportion of quality indicators of GOC conversations achieved was in Supplementary Figure 1. To facilitate comparison with the results based on the linear regression in the original ITS analysis, average marginal effects were derived from the beta regression model and back-transformation applied. Results of the beta regression and those based on linear regression were summarised in Supplementary Table 1. The estimated changes on the level and trend of average composite score after MD-PALS implementation from both models were similar.

1. Smithson, M., & Verkuilen, J. (2006). A better lemon squeezer? Maximum-likelihood regression with beta-distributed dependent variables. *Psychological methods, 11*(1), 54-71.
