# Supplementary material for: Implementation of a Multi-Disciplinary Team and Quality of Goals of Care Discussions in Palliative Surgical Oncology Patients
Source: Ann Surg Oncol. 2023 Sep 6;30(13):8054–60. doi: 10.1245/s10434-023-14190-z (PMC10625938; doi:10.1245/s10434-023-14190-z)
Supplement: Supplementary file 2 — Supplementary file2 (DOCX 13 kb) [file 10434_2023_14190_MOESM2_ESM.docx]

Supplementary Table 1. Estimated effects of MD-PALS implementation on average composite score of quality of GOC conversations

| Model | Change in level of composite score  after MD-PALS implementation | Change in trend of composite score  after MD-PALS implementation |
| --- | --- | --- |
| Linear regression  (original analysis) | Average quarterly composite score increased by  1.93 points (95% CI, 0.96, 2.90) | Change in average composite score for each additional quarter dropped by  0.08 points (95% CI, -0.41, 0.25) |
| Beta regression  (sensitivity analysis) | Average monthly composite score increased by  1.76 points (95% CI, 1.24, 2.32) | Change in average composite score for each additional month dropped by  0.004 points (95% CI, -0.08, 0.07) |
