# Supplementary material for: Implementation of a Multi-Disciplinary Team and Quality of Goals of Care Discussions in Palliative Surgical Oncology Patients
Source: Ann Surg Oncol. 2023 Sep 6;30(13):8054–60. doi: 10.1245/s10434-023-14190-z (PMC10625938; doi:10.1245/s10434-023-14190-z)
Supplement: Supplementary file 3 — Supplementary file3 (DOCX 17 kb) [file 10434_2023_14190_MOESM3_ESM.docx]

Supplementary Table 2. Exploratory subgroup analysis of pre-operative goals of care conversation by whether patient received palliative surgery

|  | Pre-  MD-PALS | |  | Post-  MD-PALS | | Odds ratio (95% CI)  (Pre-MD-PALS  as reference) | Interaction  p^/1^ |
| --- | --- | --- | --- | --- | --- | --- | --- |
|  | E | N |  | E | N |  |  |
| Deliberated on goals of surgery |  |  |  |  |  |  | 0.46 |
| No surgery | 2 | 2 |  | 15 | 15 | 6.2 (0.1,696.2) |  |
| Surgery | 31 | 42 |  | 67 | 67 | 49.3 (2.8,882.2) |  |
|  |  |  |  |  |  |  |  |
| Deliberated on patient’s prognosis |  |  |  |  |  |  | 0.97 |
| No surgery | 1 | 2 |  | 11 | 15 | 2.8 (0.1,55.2) |  |
| Surgery | 12 | 42 |  | 34 | 67 | 2.6 (1.1,5.9) |  |
|  |  |  |  |  |  |  |  |
| Deliberated on patient’s priorities &  preferences on treatment options |  |  |  |  |  |  | 0.12 |
| No surgery | 2 | 2 |  | 12 | 15 | 0.7 (0.01,35.8) |  |
| Surgery | 10 | 42 |  | 57 | 67 | 17.0 (6.4,44.6) |  |
|  |  |  |  |  |  |  |  |
| Discussed resuscitation code status |  |  |  |  |  |  | 0.31 |
| No surgery | 0 | 2 |  | 2 | 15 | 0.9 (0.02,49.2) |  |
| Surgery | 1 | 42 |  | 16 | 67 | 8.9 (1.6,50.5) |  |
|  |  |  |  |  |  |  |  |

E, no. of patients with yes response; N, total no. of patients; CI, confidence interval

/1 Interaction between MDPALS patient group and whether patient received surgery
