# Supplementary figures and images for: Implementation of a Multi-Disciplinary Team and Quality of Goals of Care Discussions in Palliative Surgical Oncology Patients
Source: Ann Surg Oncol. 2023 Sep 6;30(13):8054–60. doi: 10.1245/s10434-023-14190-z (PMC10625938; doi:10.1245/s10434-023-14190-z)

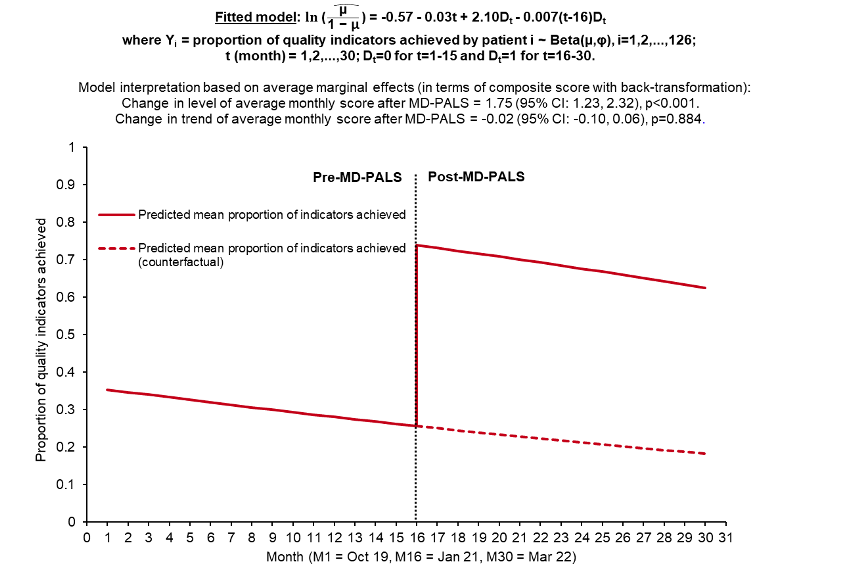

Supplement: Supplementary file 4 — Supplementary file4 (JPG 17 kb) [file 10434_2023_14190_MOESM4_ESM.jpg]
